# Feasibility of Identifying Aerobic‐Fitness Responses to Aerobic Exercise in Alzheimer's Disease: The FIT‐AD SMART Trial

**DOI:** 10.1002/alz70858_098678

**Published:** 2025-12-24

**Authors:** Fang Yu, Michael Todd, Molly Maxfield, Rodney Joseph, Jeremy J. Pruzin, Yi Su, Danni Li, David W. Coon

**Affiliations:** ^1^ Arizona State University, Phoenix, AZ, USA; ^2^ Banner Alzheimer's Institute, Phoenix, AZ, USA; ^3^ University of Minnesota, Minneapolis, MN, USA

## Abstract

**Background:**

Improvements in cardiorespiratory fitness following moderate‐intensity continuous aerobic training (MICT) are well‐documented but show variability across individuals. This heterogeneity among older adults may contribute to inconsistent findings regarding the effects of MICT on cognition in individuals with Alzheimer's disease (AD).

**Methods:**

This presentation will examine the feasibility of using aerobic fitness, as measured by peak oxygen consumption (VO2peak) through a symptom‐limited cycle‐ergometer test, to identify non‐responders to MICT and adapt exercises from the FIT‐AD Sequential, Multiple Assignment, Randomized Trial (SMART). FIT‐AD SMART aims to enroll 108 older adults with early AD, assigning 72 to MICT and 36 to stretching exercises for six months. After three months, VO2peak is reassessed to identify non‐responses, defined as <5% improvement in VO2peak from baseline. Non‐responders are re‐randomized to either high‐intensity interval training or a combined aerobic and resistance exercise program, while responders continue with MICT for another three months.

**Results:**

As of January 2, 2025, 19 participants had passed the 3‐month assessment period. They were 70.95±5.75 years of age, had 16.05±2.74 years of education, and scored 22.74±3.51 on the Montreal Cognitive Assessment (MoCA). About 57.1% of the participants were female, and 28.6% were Hispanic. Their VO2peak averaged 18.72±4.75 mL/kg/min at baseline and 21.73±6.91 mL/kg/min at three months, representing a mean improvement of 6.93%±10.43%. Among the 19 participants, 13 were randomized to MICT and 11 completed 3‐month exercise testing. The 11 MICT participants’ VO2peak was 19.05 mL/kg/min at baseline and 20.40 mL/kg/min at 3 months, representing a 15.2% increase. Six of the MICT participants were classified as non‐responders (VO2peak change: ‐2.96%±8.85%), and five were responders (VO2peak change: 36.9%±51.9%). In contrast, VO2peak of the stretching group (*n* = 6) decreased 6.2%.

**Conclusion:**

The findings indicate that VO2peak did not improve among half of participants assigned to MICT following 3‐months of training, although the amount of improvement was considerable among responders. These findings underscore the importance of using VO2peak as a key physiological marker for identifying exercise non‐response in older adults with AD early during exercise interventions. Subsequent tailoring of exercise interventions following non‐response to MICT may help maximize the benefits of aerobic exercise on cognitive health.

